# Cavotricuspid Isthmus-Dependent Atrial Flutter. Beyond Simple Linear Ablation

**DOI:** 10.31083/j.rcm2501011

**Published:** 2024-01-09

**Authors:** Julian Abdala-Lizarraga, Javier Quesada-Ocete, Blanca Quesada-Ocete, Javier Jiménez-Bello, Aurelio Quesada

**Affiliations:** ^1^School of Doctorate, Catholic University of Valencia San Vicente Mártir, 46001 Valencia, Spain; ^2^Arrhythmia Unit, Cardiology Service, General University Hospital Consortium of Valencia, 46014 Valencia, Spain

**Keywords:** atrial flutter, cavotricuspid isthmus, catheter ablation, electrophysiology

## Abstract

The demonstration of a peritricuspid circular movement with a zone of slow conduction in the cavotricuspid isthmus, together with the high efficacy of linear ablation and widely accepted acute endpoints, has established typical flutter as a disease with a well-defined physiopathology and treatment. However, certain aspects regarding its deeper physiopathology, ablation targets, and methods for verifying the results remain to be clarified. While current research efforts have primarily been focused on the advancement of effective ablation techniques, it is crucial to continue exploring the intricate electrophysiological, ultrastructural, and pharmacological pathways that underlie the development of atrial flutter. This ongoing investigation is essential for the development of targeted preventive strategies that can act upon the specific mechanisms responsible for the initiation and maintenance of this arrhythmia. In this work, we will discuss less ascertained aspects alongside the most widely recognized general data, as well as the most recent or less commonly used contributions regarding the electrophysiological evaluation and ablation of typical atrial flutter. Regarding electrophysiological characteristics, one of the most intriguing findings is the presence of low voltage zones in some of these patients together with the presence of a functional, unidirectional line of block between the two vena cava. It is theorized that episodes of paroxysmal atrial fibrillation can trigger this line of block, which may then allow the onset of stable atrial flutter. Without this, the patient will either remain in atrial fibrillation or return to sinus rhythm. Another of the most important pending tasks is identifying patients at risk of developing post-ablation atrial fibrillation. Discriminating between individuals who will experience a complete arrhythmia cure and those who will develop atrial fibrillation after flutter ablation, remains essential given the important prognostic and therapeutic implications. From the initial X-ray guided linear cavotricuspid ablation, several alternatives have arisen in the last decade: electrophysiological criteria-directed point applications based on entrainment mapping, applications directed by maximum voltage criteria or by wavefront speed and maximum voltage criteria (omnipolar mapping). Electro-anatomical navigation systems offer substantial support in all three strategies. Finally, the electrophysiological techniques to confirm the success of the procedure are reviewed.

## 1. Introduction

Cavotricuspid isthmus (CTI)-dependent atrial flutter is a common cardiac arrhythmia characterized by the occurrence of regular, rapid atrial depolarizations of constant amplitude and morphology at a rate greater than or equal to 240 beats per minute, usually secondary to macroreentry in the right atrium [1].

The epidemiology of atrial flutter (AFL) is not known with certainty, as atrial flutter and atrial fibrillation (AF) can coexist. Atrial flutter is estimated to account for 5% to 20% of all cardiac arrhythmias diagnosed worldwide [2]. It has been reported that almost 60 million individuals globally are affected by AF and/or atrial flutter. Despite being a relatively common arrhythmia, the prevalence of atrial flutter is much lower than that of AF [3].

In Spain, atrial flutter ablations account for 22% of all ablations recorded in the national ablation registry, making it the second most commonly performed procedure after pulmonary vein ablation [4].

Patients with atrial flutter are at increased risk for heart failure, stroke, and all-cause mortality [5, 6]. There are prognostic differences between atrial flutter and atrial fibrillation. Previous studies have shown that the rate of adverse events in patients with atrial flutter is lower than in patients with atrial fibrillation, but still higher than in healthy individuals without cardiac arrhythmias [5, 6, 7].

The demonstration of a peritricuspid circular movement with a zone of slow conduction in the CTI, together with the high efficacy of linear ablation with widely accepted acute endpoints, has established typical flutter as a disease with a well-defined physiopathology and treatment [1, 2, 5]. However, certain aspects regarding its deeper physiopathology, ablation targets, and methods for verifying the results remain to be clarified. In this work, we will discuss less ascertained aspects alongside the most widely recognized general data, as well as the most recent or less commonly used alternatives regarding the electrophysiological evaluation and ablation of typical flutter.

## 2. Classification

The classification of atrial flutter has evolved over the last few years. Initially, it was categorized as a common or uncommon flutter based on F-wave morphology. Subsequently, the classification of atrial flutter was updated to include type I and type II, based on the frequency of atrial activation. However, both classifications are no longer in use.

In 2001, the Working Group of Arrhythmias of the European Society of Cardiology and the North American Society of Pacing and Electrophysiology proposed a new classification of atrial flutter that is still in use today [8]. This classification system divides atrial flutter into two main types based on electrocardiographic characteristics and the circuit that sustains the arrhythmia: typical atrial flutter and atypical flutter.

### 2.1 Typical Atrial Flutter

**Fig. 1.**
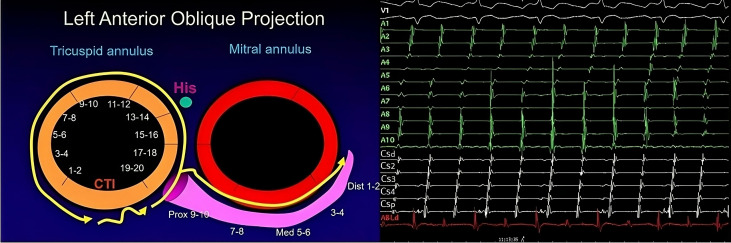
**Counterclockwise typical flutter activation**. Left: 1-2 to 19-20 represents the 10 dipoles from a 20-poles catheter placed in the right atrium close the tricuspid valve. Prox 9-10 to Dist 1-2 represents the 5 dipoles from a 10-poles catheter inserted into coronary sinus, around mitral annulus. Yellow arrows show wavefront propagating through the interatrial septum, right atrium roof, and lateral wall of the right atrium before returning to the cavotricuspid isthmus (CTI) with the slow conduction zone. Left atrium is activated passively from this wavefront crossing interatrial septum in a *caudocraneal* sense. Right: Electrophysiological tracings from these catheters. A1–A10 represents the bipolar recordings of the 20-poles catheter displaying consecutive, sequential activation from A10 to A1, followed by the electrogram recorded by the ablation catheter (ABLd) located in the CTI. From there, the activation spreads both toward the left atrium, electrograms registered by coronary sinus (CS) catheter, CSd to CSp, and ascending again through the septum (A10). CTI, cavotricuspid isthmus; His, His bundle; Prox, proximal; Med, medium; Dist, distal; ABLd, distal ablation catheter; CSd, distal coronary sinus; CSp, proximal coronary sinus.

**Fig. 2.**
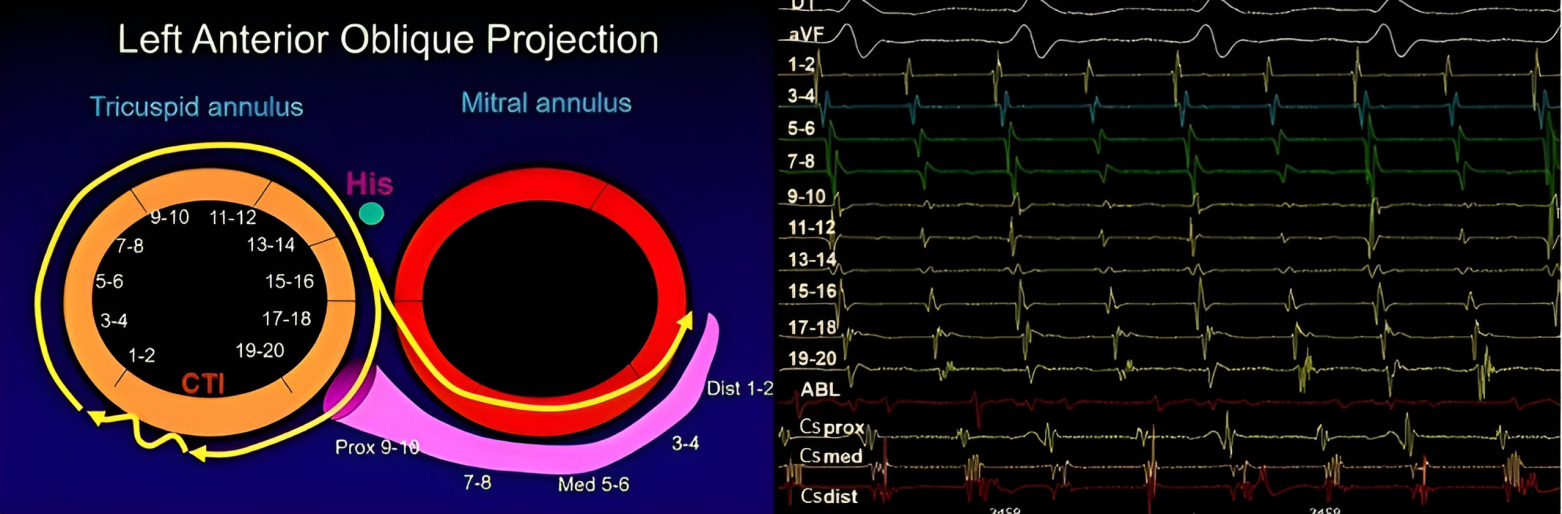
**Reverse typical flutter**. The arrangement of the catheters, bipoles and recordings is the same than in Fig. 1. (Left) Scheme illustrating how the activation front in this case moves in a clockwise direction, propagating through the anterolateral wall of the right atrium in a caudocranial direction before reaching the septal wall in a craniocaudal direction. (Right) Right atrium electrograms shows the clockwise sequence, starting at A1, low anterolateral wall, and propagating along contiguous dipoles toward right atrium roof (pairs 7-8 to 11-12), septum and cavotricuspid isthmus (CTI) where the ablation catheter (ABL) is placed. His, His bundle; Prox, proximal; Med, medium; Dist, distal; CS, coronary sinus.

Characterized by a reentrant circuit originating from the CTI and is presently the most prevalent macroreentrant arrhythmia. Depending on the direction of depolarization, it can be subclassified as counterclockwise, when the activation front passes through the CTI reaching the septal wall in a caudocranial direction and finally the anterolateral wall of the right atrium in a craniocaudal direction. Alternatively, it is referred to as clockwise or reverse when the activation front travels in the opposite direction, which is less common (Figs. 1,2).

### 2.2 Atypical Flutter

A type of atrial macroreentrant tachycardia that does not rely on the cavotricuspid isthmus circuit. Instead, this circuit can be located in either the right or left atrium. Activation of the circuit often occurs around a surgical scar, after ablation, or even spontaneously (idiopathically). As a result, atypical flutter usually affects patients with a history of cardiac surgery, previous ablation, congenital heart disease, atrial fibrosis or cardiomyopathy [9]. Common localizations are perimitral and roof-dependent left atrial flutter.

It should be noted that previous procedures (surgery, ablation), drugs (class IC antiarrhythmics) or structural heart disease can cause atypical findings in patients with atrial flutter and, on the other hand, some left atrial flutters can mimic typical flutter electrocardiographic morphology.

## 3. Electrophysiological Features

In general, conditions that must be met for a reentrant mechanism to be initiated, include the presence of a circuit, a unidirectional conduction block, and a slow conduction zone. It is estimated that macroreentry in the right atrium around the tricuspid annulus accounts for approximately 75–80% of all cases of atrial flutter [1].

The flutter circuit has two barriers that favor the genesis and maintenance of the arrhythmia: the posterior barrier of the activation wave is determined by the crista terminalis and its continuation with the Eustachian valve, while the anterior barrier is determined by the tricuspid annulus [10].

The CTI plays a crucial role in the propagation of electrical impulses in atrial flutter, serving as the slow conduction zone of the circuit and therefore being highly vulnerable to interval-dependent conduction block [11]. The effectiveness of antiarrhythmic drugs in terminating atrial flutter can be partly attributed to the interruption of conduction through this critical zone of the circuit. However, atrial flutter is often difficult to treat pharmacologically.

In typical counterclockwise flutter, the circuit involves the CTI, peritricuspid as described previously. Conversely, in typical clockwise flutter, the activation wave follows the reverse path [12] (Figs. 1,2).

The main electrophysiological characteristics of atrial flutter can be summarized as a reentrant arrhythmia with an excitable gap that can be transiently entrained or terminated by rapid atrial pacing. As a reentrant arrhythmia, once an initial zone in the right atrium is activated, the wavefront spreads through an area of slow conduction that allows time for the initial atrial tissue to regain excitability, which is then re-activated. The excitable gap, which is the part of the reentrant circuit that has regained its excitability, can be depolarized again resulting in the propagation of the atrial impulse. For this reason, atrial flutter can be initiated, triggered, or terminated by an extra stimulus or rapid atrial pacing.

In the majority of patients with atrial flutter, the wavefront propagates in an anterior direction towards the superior vena cava [13]. Nonetheless, previous studies have demonstrated that the circuit may involve the posterior wall of the right atrium in certain patients [14]. This finding suggests that the crista terminalis does not always act as a fixed conduction barrier and that the circuit may be located posterior to the superior vena cava.

Under normal conditions, atrial myocardial fibers have a low resistance and a longitudinal conduction velocity that is greater than the transverse conduction velocity [15]. Anisotropy, defined as the property of tissues to conduct the electrical impulse in different directions at different rates, is an important feature of the crista terminalis because it acts as a functional barrier by allowing faster conduction in the longitudinal direction than in the transverse direction [16]. In the case of typical flutter, the anisotropy of conduction secondary to the direction of the myocardial fibers in the crista terminalis and the CTI are two determining factors in allowing and maintaining the reentrant mechanism by forcing activation through the roof of the right atrium or in the upper and posterior part of the right atrium [17].

In some cases where the atrial rate is lower, transverse conduction through the crista terminalis may be possible [18]. Similarly, re-entry through the CTI may be observed in patients who have undergone CTI ablation [19]. Furthermore, the administration of antiarrhythmic drugs can increase anisotropy, which would explain the development of atrial flutter in some patients with atrial fibrillation [20].

Interestingly, studies have demonstrated that the thickness of the crista terminalis is increased in patients with atrial flutter compared to those with atrial fibrillation, and this may contribute to its ability to block transverse conduction in the right atrium [21]. These findings suggest that the crista terminalis plays an important role in the pathophysiology of atrial flutter.

Some authors support the hypothesis that a fundamental feature determining the genesis and maintenance of atrial flutter is the presence of a functional, unidirectional line of block between the two vena cava. It is theorized that episodes of paroxysmal atrial fibrillation can trigger this line of block, which may then allow the onset of stable atrial flutter. Without this unidirectional line of block, the patient will either remain in atrial fibrillation or return to sinus rhythm [22].

Electrophysiological studies have revealed the presence of low voltage electrograms and areas of slow conduction in the right atrium, particularly in the CTI [23]. These findings have been described as markers of atrial remodeling with arrhythmogenic potential in patients with atrial flutter and are directly proportional to the burden and duration of the arrhythmia [24]. This atrial remodeling is responsible for the electrophysiological changes that happen in patients even in sinus rhythm, such as shortening of the action potential and effective refractory period, which are associated with increased susceptibility to induction of atrial tachyarrhythmias by atrial extrasystoles. Unlike patients with atrial fibrillation, these changes are not attributed to increased fibrosis or inflammation of the atrial tissue [24].

Patients with atrial flutter often exhibit many characteristic findings that are thought to be secondary to atrial remodeling, including increased right atrial dilatation, prolonged P wave duration on the electrocardiogram, sinus node dysfunction, and decreased voltage and conduction velocity of the electrical impulse in electrophysiological study [25].

## 4. Risk Factors

A clear association has been observed between atrial flutter and *patient gender*, as approximately 80% of individuals diagnosed with this arrhythmia are men [26]. In addition, there are many known risk factors for developing atrial flutter, including advanced age, alcohol consumption, arterial hypertension, diabetes, chronic obstructive pulmonary disease, and engaging in high-intensity sports [27].

This arrhythmia usually arises in patients with some degree of *structural heart disease*; however, 15–20% of cases that develop atrial flutter have no apparent structural heart disease [1, 26]. Typical flutter is found to be more prevalent in individuals both with or without structural heart disease compared to atypical flutter. Structural abnormalities such as atriotomy scars, patch closure of atrial septal defects, repaired congenital heart disease, or suture lines after cardiac surgery can act as barriers to the conduction of electrical impulses and promote the development of a reentrant circuit [28]. Some echocardiographic studies suggest that left atrial dilatation may predict the development of atrial flutter or atrial fibrillation [29].

The use of certain *medications* like flecainide, propafenone, dronedarone, or amiodarone in patients with atrial fibrillation may lead to the development of atrial flutter in up to 3.5 to 15% of cases [30].

Atrial flutter can manifest during the *perioperative period of cardiac surgery* or as a late complication. In some cases, ablation of a left atrial arrhythmia may act as a substrate for the onset of left atrial flutter [31].

In addition to the previously mentioned risk factors, *other less common factors* that have been associated with an increased risk of atrial flutter, include obesity, thyrotoxicosis, sleep apnea, acute pericarditis, pulmonary disease, pulmonary thromboembolism, and occurrence after acute myocardial infarction [32].

## 5. Clinical Manifestations

Atrial flutter is mainly associated with symptoms such as palpitations, dyspnea, asthenia and to a lesser extent, chest pain, syncope and hypotension. These clinical manifestations are typically a result of the increased ventricular response associated with atrial flutter. Hemodynamically, the increase in ventricular rate leads to a rise in atrial pressure, a decrease in ventricular end-diastolic pressure, and thus a decrease in systolic pressure and an increase in diastolic pressure without any change in the cardiac index [33].

It is important to note that patients with atrial flutter may develop serious secondary complications, such as heart failure, myocardial ischemia, tachycardia-induced cardiomyopathy, stroke, or systemic embolism [34]. Therefore, early detection and management of atrial flutter are essential to prevent such adverse outcomes.

## 6. Electrocardiographic Features

**Fig. 3.**
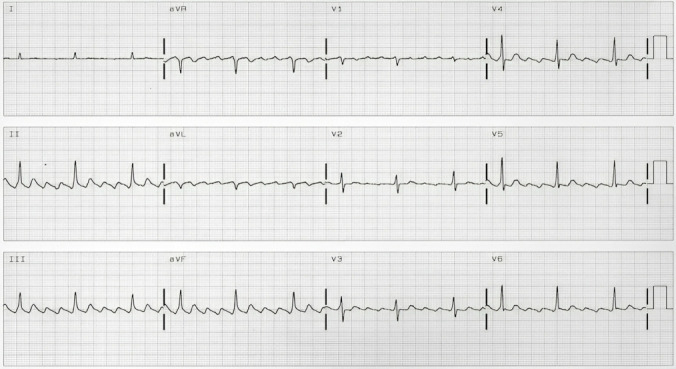
**Electrocardiogram of a typical counterclockwise flutter**. Negative F waves in the inferior leads and positive in V1 with no isoelectric line between them.

Given that the activation wave moves from the inferior to the superior right atrium septum, typical counterclockwise flutter is characterized electrocardiographically by the presence of negative waves with a slow downward slope followed by a rapid upward slope in the inferior leads and positive in V1 (coinciding with craneocaudal lateral wall activation) with no isoelectric line between them. These waves, known as “F” waves, have a cycle length between 250–170 milliseconds (240–350 beats per minute) (Fig. 3). In contrast, typical clockwise flutter is characterized by an inverted electrocardiographic pattern with positive waves in the inferior leads and negative waves in V1. Both patterns have been described as simulating a “sawtooth” pattern and are particularly evident in DII, DIII, and aVF [35]. These characteristic findings may be altered in patients who have undergone cardiac surgery, catheter ablation or who are treated with antiarrhythmic drugs [36].

Lai *et al*. [37] have proposed a novel electrocardiographic criterion to distinguish clockwise from counterclockwise flutter by assessing the ratio of F-wave amplitudes in leads DI and aVF. In patients with counterclockwise flutter, this ratio is typically greater than 2.5, while patients with clockwise flutter exhibit a ratio of less than 2.5.

The electrocardiogram of atypical atrial flutter is identified by the following features: absence of P waves, presence of flutter waves with uniform morphology; unlike typical atrial flutter, an isoelectric line may be visible in some cases if conduction is significantly delayed, and in certain cases, patients with atypical atrial flutter may display concordance in the polarity of F waves in both inferior leads and V1.

Under normal physiological conditions, conduction through the atrioventricular node typically occurs with a 2:1 ratio. However, in patients receiving group I antiarrhythmics such as flecainide or propafenone, a 1:1 ratio of atrioventricular conduction may be observed.

The electrocardiographic diagnosis of flutter can be difficult to establish in some cases, especially when 1:1 atrioventricular conduction is present. In this regard, intravenous administration of adenosine can be helpful as it increases the degree of atrioventricular block, which enables better visualization of typical F waves on the surface electrocardiogram [38].

It is important to emphasize that the electrocardiographic diagnosis of flutter should be based on atrial activity, not ventricular activity, since different degrees of atrioventricular block may cause irregular ventricular rates.

The presence or absence of an isoelectric line on the 12-lead electrocardiogram may help when planning ablation for atrial flutter. The presence of an isoelectric interval in all leads indicates the presence of a narrow, slow-conducting isthmus that may be treatable with focal ablation. In these cases, ablation could be attempted after identifying the isthmus without the need to fully define the tachycardia circuit. However, in the absence of an isoelectric line, a detailed activation map combined with entrainment techniques is necessary to define the circuit and plan the most effective linear ablation strategy.

Some authors have proposed a systematic classification of flutter based electrocardiographic F waves characteristics. Milliez *et al*. [39] proposed classification of counterclockwise typical atrial flutter into three groups: Type 1 is characterized by the presence of completely negative F waves in inferior leads; Type 2, displaying small positive deflections and Type 3, shows wide terminal positive deflections in the inferior leads. The latter two groups have been associated with a higher incidence of left atrial dilatation, heart disease and atrial fibrillation compared to patients in the first group.

This working group also proposed a classification system for clockwise atrial flutter, which comprises two groups: Type 1, exhibiting narrow-based, positive F waves with a clear isoelectric line; and Type 2, displaying broad-based F waves with positive and negative components and a short or absent isoelectric segment.

### Vectorcardiographic Analysis of Atrial Loops for Characterisation of Typical and Atypical Atrial Flutter and Body Surface Potential Recordings

Other ECG-derived techniques have been proposed to enhance the differentiation between typical and atypical forms. Particularly noteworthy among these methods is the vectorcardiogram (VCG) [40, 41, 42], along with body surface potential mapping (BSPM) [43, 44].

Regarding the VCG, this technique has been acknowledged within the medical community for decades [45]. VCG explores the loop depicting the sequence of atrial activation vectors and has shown a promising capability for distinguishing between different flutter patterns [42].

Furthermore, the assessment of the vectorcardiogram loop trajectory, acquired through the application of Dower’s Inverse Transform to the 12-lead electrocardiogram, demonstrated a potential improvement in distinguishing typical from atypical atrial flutter. Our investigation [40] identified that incorporating parameters such as global trajectory, pathway complexity, and distance could increase the precision of discrimination. In addition, an intrapatient analysis revealed greater stability in the VCG loops associated with typical AFL in contrast to the variability observed in cases of atypical AFL.

BSPM utilizes a specialized vest equipped with more than 60 strategically positioned electrodes, covering the patient’s torso and limbs. These electrodes capture signals that undergo sophisticated computational analysis, resulting in the creation of isochronous lines. This technique provides valuable information about the direction and rotation of atrial activation [43] and has been shown to improve the detection and classification of typical atrial AFL.

Refining discrimination can be accomplished through the implementation of advanced processing methodologies, exemplified by the utilization of phase maps [46, 47]. In this context, phase refers to the precise point within an oscillation cycle that a signal occupies at a specific moment. Phase mapping entails a comprehensive analysis of signal oscillations across their complete temporal span, irrespective of their amplitude [47], thereby enhancing the precision of differentiation. Hence, analysis of phase changes over space provides information on the patterns of organization (repetitive activity) and could help to assess their stability in time and space [44].

**Fig. 4.**
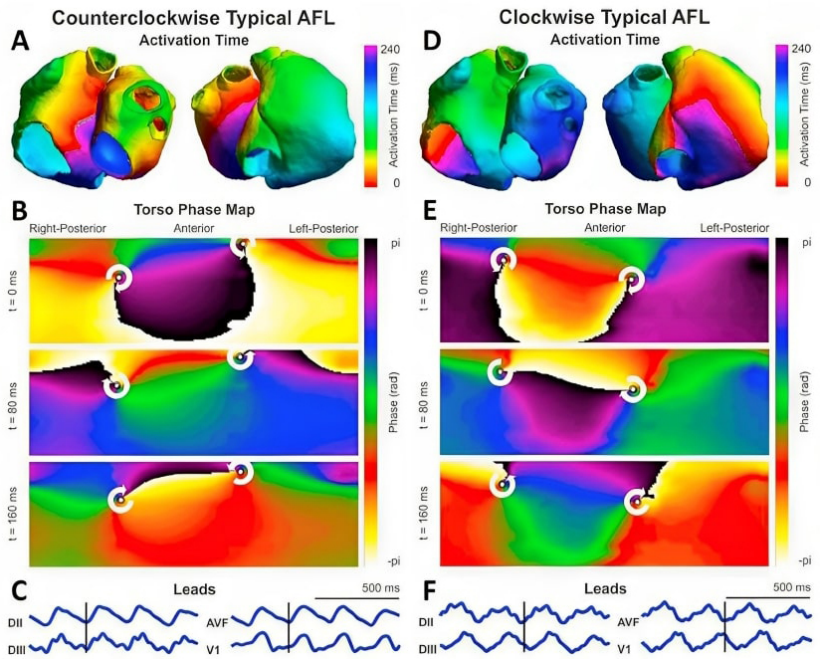
**Use of phase maps to discriminate between typical atrial flutter (peritricuspid macroreentrant, left panel) and other reentrant tachycardia around pulmonary veins (right panel)**. (A,D) Isochronal maps in the atria illustrating macro-reentrant behaviors. (B,E) Surface phase maps at three consecutive time moments in each tachycardia. (C,F) Most representative standard derivations in AFL. AFL, atrial flutter. White arrows = singularity points. From: Liberos A, Rodrigo M, Hernandez-Romero I, Quesada A, Fernandez-Aviles F, Atienza F, Climent AM, Guillem MS. Phase singularity point tracking for the identification of typical and atypical flutter patients: A clinical-computational study. Comput Biol Med. 2019; 104: 319–328 [46].

We have studied the surface representation of the macro-reentrant activity by meticulously monitoring singularity points within surface phase maps derived from band-pass filtered body surface potential mappings. Spatial distribution of singularity point showed significant differences between typical and atypical AFL (Fig. 4, Ref. [46]), suggesting phase maps are a promising tool for the noninvasive characterization of the flutter circuit, and thus, for AFL ablation planning [46].

## 7. Treatment

There are three main pillars that must be considered when managing patients with atrial flutter. These include anticoagulation therapy to prevent the occurrence of embolic events, management of ventricular response to improve symptoms and avoid the development of heart failure and rhythm control to restore and maintain sinus rhythm.

### 7.1 Anticoagulation

There is limited information on the true incidence of systemic embolism or the benefits of anticoagulation therapy in patients with atrial flutter. In a systematic review, the calculated risk of thromboembolic events in patients with persistent flutter was 3% per year, similar to the risk for atrial fibrillation [48]. In addition, a significant proportion of patients diagnosed with atrial flutter, experience intermittent episodes of atrial fibrillation, which complicates the precise determination of the associated thromboembolic risk in such cases. For this reason, it has been decided to use the same preventive measures as for patients with atrial fibrillation.

Patients diagnosed with atrial flutter show an increased incidence of intracardiac thrombus formation within the left atrial appendage [49], as do patients with atrial fibrillation. Thromboembolic risk factors described in patients with atrial flutter include those reported for atrial fibrillation, such as valvular pathology, prosthetic valves, advanced age, left ventricular systolic dysfunction, heart failure, hypertension, diabetes, vascular disease, and a history of previous thromboembolic events [26]. Although rare, some patients with atrial flutter have no identifiable embolic risk factor [50].

Additionally, as mentioned before, an unsolved problem is the observed strong association between atrial fibrillation and atrial flutter. Approximately half of the patients diagnosed with atrial flutter develop atrial fibrillation during follow-up, including those who have undergone CTI ablation [5]. However, previous studies have shown that the rate of adverse events in patients with atrial flutter is lower than in patients with atrial fibrillation, but still higher than in healthy individuals without cardiac arrhythmias [6]. Notably, the risk of stroke is lower in patients with isolated atrial flutter but it is increased in those who subsequently develop atrial fibrillation [7].

Anticoagulation therapy is indicated in patients with atrial flutter whose CHA2DS2-VASc score is 2 or greater in males and 3 or greater in females [51]. It is recommended that anticoagulation be initiated when the CHA2DS2-VASc score is 1 in males and 2 in females, always considering the patient preferences and benefits of anticoagulation in these patients [52, 53].

Vitamin K antagonists (VKAs), mainly warfarin, have been shown to significantly reduce stroke risk by 64% and mortality by 26% when compared to control or placebo [54]. VKAs represent the only treatment option with established safety in patients with moderate/severe rheumatic mitral valve disease and/or individuals with a mechanical prosthesis [51].

However, the use of VKAs is limited by their narrow therapeutic range and, especially, by the lack of dose-effect relationship, due to their metabolism and the huge number of pharmacological and non-pharmacological interactions, required for frequent monitoring of international normalized ratio (INR) levels and regular dose adjustments. Direct oral anticoagulants (DOACs) were developed to overcome these limitations, and in their pivotal trials, all four DOACs have proven to be non-inferior to warfarin in preventing stroke/systemic embolism. Additionally, DOACs have been associated with significant reductions in hemorrhagic stroke and all-cause mortality, as well as a similar risk reduction for ischemic stroke when compared to VKAs. However, it is important to consider that DOACs have been linked to an increased risk of gastrointestinal bleeding (excluding low-dose dabigatran and apixaban) [55]. DOACs therapy is generally associated with higher adherence rates compared to VKAs, mainly due to the improved pharmacokinetic profile of DOACs [56] and their favorable safety and efficacy.

Discontinuation of anticoagulation after ablation of typical flutter remains an unresolved issue. In the absence of concomitant untreated atrial fibrillation and if CHA2DS2-VASC is not elevated, it is common to discontinue anticoagulation, especially VKAs because of their limitations in daily life. However, in a recent meta-analysis [57], a non-negligible risk of stroke after ablation was found (ranging from 1 to 10%, mostly on VKAs). Remarkably no differences were detected between patients with and without anticoagulation. Thus, the decision to discontinue anticoagulation should remain individualized, taking into account scores such as CHA2DS2-VASC and HAS-BLED, comorbidities, presence of other structural heart disease or the treatment’s impact on the patient’s lifestyle, and maintaining long-term monitoring for flutter recurrence or the appearance of AF [57].

In cases where a rhythm control strategy is chosen, anticoagulation may be omitted if the duration of the flutter episode is less than 48 hours. However, in patients with episodes of longer duration, it is essential to initiate anticoagulation therapy for at least 3 weeks before cardioversion or to rule out the presence of left atrial thrombi via transesophageal echocardiography [58, 59].

Following cardioversion of atrial flutter, it is recommended to maintain anticoagulation therapy for 4 weeks in patients with low thromboembolic risk and chronically in high risk individuals [60]. In patients undergoing CTI ablation, anticoagulation should be maintained for at least 2 months after the procedure [51]. This indication is based on the increased risk of thrombus formation due to the left atrial stunning that occurs after cardioversion or CTI ablation. However, there is currently insufficient evidence from randomized trials to establish a single strategy for stopping anticoagulation in patients after CTI ablation.

### 7.2 Rate Control (Targets, Drugs and Atrioventricular Node Ablation)

Atrial flutter is characterized by a suboptimal response to pharmacological treatment when compared to atrial fibrillation [51]. Indications for considering a rate control strategy in patients with atrial flutter include:

- Acute control of ventricular response to reduce or relieve symptoms during a first or recurrent episode in patients with persistent flutter.

- Chronic control of the ventricular response to prevent the onset of symptoms in patients with recurrent flutter who are not eligible for CTI ablation.

- Prevention of the development of arrhythmia-induced cardiomyopathy in patients with atrial flutter who are not considered suitable candidates for ablation procedures.

Heart rate goals for patients with atrial flutter have been extrapolated from atrial fibrillation studies, with a rate inferior to 110 beats per minute as a therapeutic goal [61].

For patients with atrial flutter who require acute heart rate control and are not candidates for electrical cardioversion, drugs such as verapamil, diltiazem, digoxin, intravenous beta-blockers, or amiodarone may be used in some cases [62, 63]. High doses or combinations of these drugs are usually required to achieve adequate heart rate reduction.

Non-dihydropyridine calcium channel blockers, such as verapamil and diltiazem, can be used for acute or chronic rate control in patients with atrial flutter [64]. It is important to note that these drugs should be avoided in patients with class III or IV heart failure and used with caution in patients with sinus node disease, second or third-degree atrioventricular block, preexcitation, arterial hypotension, or those taking medications that depress sinus or atrioventricular (AV) node function.

Most of the evidence supports the use of intravenous beta-blockers, particularly esmolol [65], as the primary therapy for acute control of ventricular rate in patients with atrial flutter. Among the longer-acting beta-blockers, atenolol, nadolol and metoprolol can be used, notably in patients with a history of coronary artery disease or bisoprolol, metoprolol and carvedilol in systolic heart failure. However, it is important to note that these drugs can have significant adverse effects, such as bronchospasm, arterial hypotension, high-grade atrioventricular block, bradycardia, or worsening of heart failure.

In very selected cases, digoxin alone or in combination with other drugs can be used for ventricular rate control. Nevertheless, the effectiveness of digoxin is comparatively lower, and there is a greater likelihood of toxicity, particularly in geriatric patients [66].

Amiodarone can be considered in this context when other rate-controlling drugs are contraindicated or do not achieve sufficient rate control, and it is typically reserved for critically ill patients [67], assuming the risk of facilitating an unwanted pharmacological cardioversion.

In cases refractory to pharmacological treatment, atrioventricular node ablation and pacemaker implantation may be an alternative [68, 69]. However, this approach is infrequently employed in patients with atrial flutter due to the high level of effectiveness and low risk of complications associated with CTI ablation. Thus, it is strictly reserved for those patients who are unresponsive or intolerant to medical treatment for rate or rhythm control and are not candidates for CTI ablation.

### 7.3 Rhythm Control

Indications for urgent rhythm control in patients with atrial flutter include both hemodynamic instability secondary to rapid ventricular response and patients with preexcitation through an accessory pathway.

Due to the high rate of recurrence of atrial flutter and the high percentage of success with a low complication rate, radiofrequency (RF) ablation is considered the therapeutic option of choice for rhythm control in patients with this arrhythmia and should be considered after the first symptomatic episode [51]. Nevertheless, exceptions to this rule exist, such as in the case of atrial flutter triggered by reversible conditions like pneumonia, hyperthyroidism, or other illnesses.

#### 7.3.1 Pharmacological Treatment

Similar to AF treatment, the drugs of first choice for maintaining sinus rhythm are antiarrhythmic class IC, in the absence of structural heart disease, or class III if it is present [51]. Special caution should be taken with the administration of antiarrhythmic drugs from the IC group, up to 25% may develop typical atrial flutter [70]. Flecainide and propafenone can slow conduction through atrial tissue more than they prolong refractoriness. This property facilitates organized AF activity and can promote the development of typical atrial flutter [71]. This condition is known as class IC atrial flutter. Characteristically, its cycle length is greater than spontaneous typical flutter and it can lead 1:1 atrioventricular conduction and thus a significant increase in ventricular response, potentially causing hemodynamic compromise or even ventricular fibrillation [70, 71]. Consequently, it is usually recommended to prescribe AV nodal-blocking agents in addition to class IC drugs to reduce this potential risk [70].

Ibutilide is commonly used as the first-line therapy for rhythm control in patients with atrial flutter, with a success rate of cardioversion to sinus rhythm of around 60% [72, 73]. This drug has been shown to be more effective than other antiarrhythmics such as amiodarone, sotalol, or procainamide in this context [74, 75, 76]. However, caution should be exercised when administering due to increased risk of QT interval prolongation and therefore of torsades de pointes.

Dofetilide is an approved class III antiarrhythmic drug available in both oral and intravenous formulations in the United States. In some studies, intravenous dofetilide has proven to be significantly more effective than both amiodarone and placebo in restoring sinus rhythm in patients with atrial fibrillation or flutter, although it is associated with an increased risk of torsade de pointes. Moreover, the efficacy of dofetilide was found to be higher in atrial flutter than in atrial fibrillation, with cardioversion rates of 75% and 22%, respectively [77, 78].

Even in patients with left ventricular dysfunction, dofetilide was able to maintain sinus rhythm in 79% of the patients within the first year, despite not affecting all-cause mortality, dofetilide demonstrated a significant reduction in mortality risk by restoring and maintaining sinus rhythm [79]. Additionally, treatment with dofetilide led to reduced rates of all-cause and congestive heart failure hospitalization [78].

#### 7.3.2 Overdrive Pacing Suppression and Cardioversion Techniques 

Atrial overstimulation is an alternative for cardioversion in patients with atrial flutter who have transitory or permanent pacing devices, with an efficacy rate of 50–90% [80]. It should be considered as a treatment option for patients with atrial flutter who have epicardial electrodes after cardiac surgery or an atrial pacing device. This technique has been shown to be more effective in postoperative flutters and in young patients without structural heart disease and can be performed without the need for sedation or anesthesia [81, 82].

It is recommended to initiate pacing at a rate of 10 beats per minute above the rate of atrial flutter, gradually increasing by 10 beats per minute until reaching a maximum rate of 400 beats per minute or until flutter interruption and the patient returns to sinus rhythm or a paced rhythm. It is important to note that atrial pacing can decrease the atrial flutter cycle length, accelerate it, or even induce atrial fibrillation [83].

Atrial pacing can also be administered from a catheter inserted into the oesophagus, although it requires higher pacing energy and is therefore usually more painful for the patient, and in some cases may induce ventricular arrhythmias [84].

Synchronized cardioversion, whether external or internal, is a highly effective and safe technique for rhythm control in patients with atrial flutter. The effectiveness of this technique in patients with atrial flutter is close to 100% [85], and the recurrence rates of arrhythmia after cardioversion are usually lower than those observed in patients with atrial fibrillation [86]. It is the preferred technique in patients with hemodynamic instability due to rapid ventricular response. The initial energy level recommended is 50 joules [or ≤100 joules], although frequently 25 J can be enough, and a biphasic direct current is preferred to increase efficacy and minimize adverse effects.

The pharmacological therapy aimed at maintaining sinus rhythm in patients who have previously experienced an episode of atrial flutter is frequently ineffective and associated with high recurrence rates [87]. The success rate for maintaining sinus rhythm following pharmacological cardioversion has been estimated to range between 20–30% per year, with a higher risk of recurrence observed in patients with heart failure and right atrial dilation [88]. Favorable prognostic factors for maintaining sinus rhythm include normal atrial size, recent onset of arrhythmia, and absence of secondary causes or heart failure [89]. Medications employed for this purpose include antiarrhythmic drugs from the IA and IC groups, beta-blockers, and amiodarone.

## 8. Electrophysiological Study and Ablation Techniques

The advantages of ablation over antiarrhythmic treatment in the rhythm control strategy have been studied in several randomized trials, with the former being superior in terms of patient quality of life, lower recurrence of flutter, and lower rate of hospitalizations or emergency visits [90, 91].

Therefore, RF ablation of the CTI is the preferred option for long-term maintenance of sinus rhythm in patients with typical atrial flutter. A meta-analysis including 21 studies showed a success rate of 91.7% following a single procedure and 97% with multiple procedures [92].

Ablation of the CTI is typically indicated in patients with atrial flutter [93] in: (a) symptomatic recurrent episodes despite medical treatment (Class I recommendation); (b) after a first symptomatic episode, especially in patients with hemodynamic instability (Class IIa); (c) persistent atrial flutter or those who develop left ventricular systolic dysfunction secondary to cardiomyopathy induced by this arrhythmia (Class I).

### 8.1 Electrophysiological Study

**Fig. 5.**
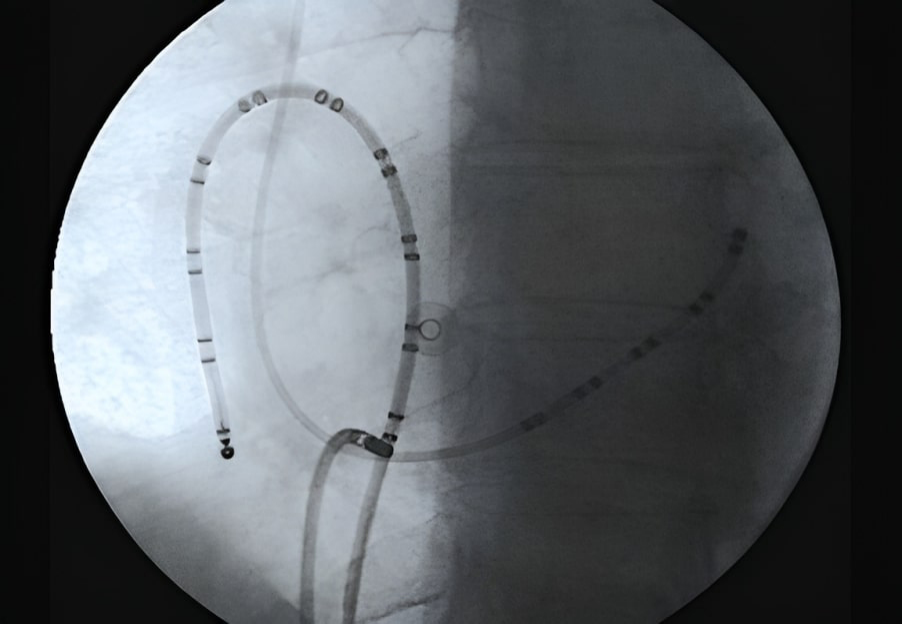
**Usual catheters positioning during a typical CTI ablation**. Left anterior oblique X-ray projection is showed. Multipolar catheters around the tricuspid valve (ten dipoles) and in the coronary sinus (5 dipoles) at the left atrioventricular groove delimiting the mitral annulus inside it. Distal part of ablation catheter is on the CTI. CTI, cavotricuspid isthmus.

During an electrophysiological study of atrial flutter, multipolar catheters are commonly used. These catheters are inserted into the right atrium forming a loop around the tricuspid valve annulus. It is important to position the catheter used for mapping the right atrium in front of the crista terminalis. Another decapolar catheter is inserted into the coronary sinus, mapping left atrial activation, and placing the distal end of the catheter in the coronary sinus can be helpful in determining the activation sequence of the arrhythmia [94] (Fig. 5).

**Fig. 6.**
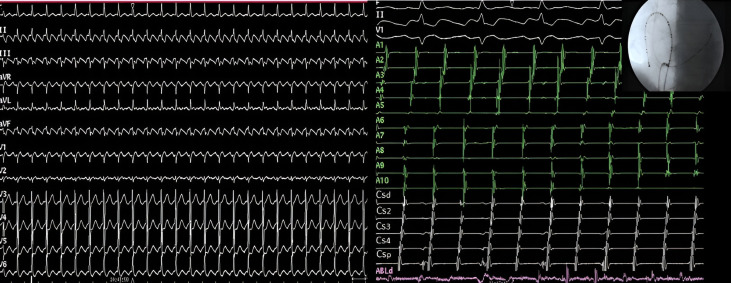
**Electrocardiographic and electrophysiological tracings in typical atrial flutter**. Left panel: Surface ECG of counterclockwise flutter with 2:1 ventricular conduction. Right panel: Endocavitary tracings of a counterclockwise flutter showing in the recordings from duodecapoles catheters relatively fast caudocranial activation of septum (pairs A10 to A7) followed by superior part of RA (A6-A5), lateral wall (A4-A1) and finally CTI (ABLd). LA is activated proximal to distally, secondarily from this circuit (coronary sinus tracings CSp to CSd). ECG, elcetrocardiogram; CTI, cavotricuspid isthmus; LA, left atrium; RA, right atirum; CSp, proximal coronary sinus; CSd, distal coronary sinus.

Ablation can be performed in sinus rhythm or during flutter (Fig. 6). If electrocardiogram (ECG) is typical and the patient has no history of cardiac surgery or previous ablations, CTI ablation can be performed directly in sinus rhythm without induction. However, in patients with previous cardiac surgery or non-completely typical ECG, it is necessary to confirm the involvement of the right atrium and the CTI in the circuit using pacing mapping techniques during spontaneous or induced atrial flutter [95, 96, 97].

By definition, macro-reentrant tachycardias such as atrial flutter can be induced and terminated by pacing. To induce typical flutter, it is recommended to stimulate the right atrium with runs of 8–10 extra stimuli, with decremental coupling intervals or increasing frequencies from 200 to 350 beats per minute or until a 2:1 capture is achieved [94]. The direction of the induced flutter, whether clockwise or counterclockwise, will depend on the permeability of the CTI and the site of pacing. Thus, pacing the lower part of the lateral wall of the right atrium facilitates counterclockwise block of the isthmus and the onset of clockwise flutter, while pacing the ostium of the coronary sinus facilitates clockwise blockage of the isthmus and the onset of counterclockwise flutter [95, 96]. Use of high pacing rates can trigger atrial fibrillation [97].

Entrainment techniques using right atrial pacing and resulting return cycles can be useful in confirming the macro-reentry mechanism and the involvement of the CTI in the circuit of atrial flutter. In the ablation of atypical flutters, entrainment techniques are frequently employed to identify conduction isthmuses between anatomical obstacles. This technique allows for precise localization of ablation points, which is crucial for the effectiveness of the procedure. In addition, these maneuvers can be useful in determining the right or left origin of the flutter. It is recommended to stimulate at rates that are slightly lower than those of the flutter (≤20 ms) in order not to interrupt or modify it. Entrainment with concealed fusion, as well as short return cycles, serve as a guide towards the location of the circuit.

**Fig. 7.**
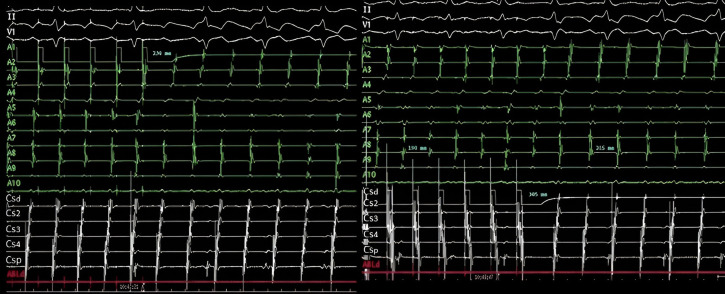
**Use of entrainment techniques to define the involvement of structures in the flutter circuit**. Left panel. Intracavitary recording during entrainment of a typical atrial flutter patient. Pacing from the low lateral wall of the right atrium (A1). Upon cessation of stimulation, the time until the next beat (postpacing interval), is 230 ms, almost identical to the cycle length of flutter (215 ms). This indicates that this point is part of the circuit of the tachycardia. Right panel. Pacing at a cycle length of 190 ms from the pair of electrodes furthest from the electrode located in the lateral wall of the left atrium. Upon cessation of stimulation, the time until the next atrial electrogram is 305 ms, notably longer than the cycle length of flutter (215 ms). As opposed to the previous example, this point is not part of the circuit and is very distant from it. Note that in the left panel, the morphology and sequence of atrial electrograms from both the 20-pole and the coronary sinus catheters are identical during entrainment and during tachycardia (concealed fusion). On the right side, they are very different, being during entrainment a mix of pacing and intrinsic beats (manifest fusion). CSp, proximal coronary sinus; CSd, distal coronary sinus.

As part of the standard entrainment protocol for atrial flutter, pacing with a cycle length less around 30 ms less than flutter one, from the CTI is commonly employed, causing transient acceleration of the flutter to the pacing rate, but without modifying the F-wave morphology in the surface electrocardiogram, nor the morphology and sequence of atrial electrograms (concealed fusion, see Fig. 7) [98]. Following pacing cessation, the atrial flutter continues at its baseline frequency, and the post-pacing interval is measured from the last pacing artifact to the first unstimulated electrogram recorded at the pacing site (Fig. 7). This interval represents the time required for the impulse to reach the circuit, complete one full revolution inside it and return to the point of pacing. If the difference between the post-pacing interval and the atrial flutter cycle length is less than 30 ms, the involvement of the CTI in the arrhythmia circuit can be ensured [99, 100].

Unlike atypical flutters where additionally to short post-pacing intervals (and concealed fusion) critical sites are required to be evaluated during electroanatomic mapping, by defining anatomic/scar boundaries with fragmented electrograms [101], in typical flutters the presence of abnormal electrograms has only been demonstrated very occasionally before ablation, in a limited number of patients [102, 103], and its significance is uncertain [104, 105, 106, 107]. The use of high-density mapping could add new data, not observed with a conventional catheter.

**Fig. 8.**
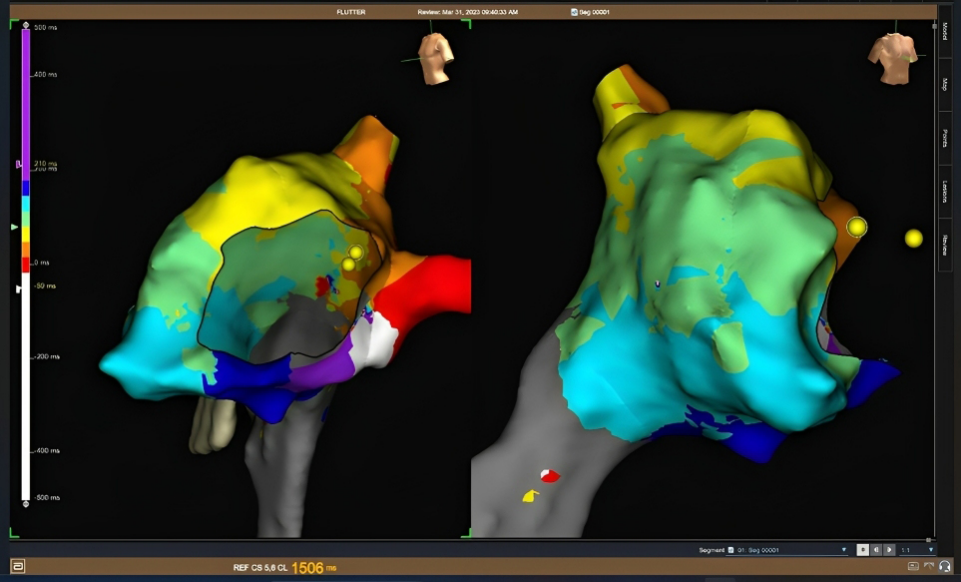
**Electroanatomic mapping of a typical counterclokwise flutter obtained with the Ensite X system**. The virtual reconstruction of the right atrium, tricuspid annulus and cava veins is shown in left anterior oblique (LAO) (left) and right anterior oblique (RAO) (right) projections. The yellow dots mark the position of the His potentials. The activation map is displayed with a colour code (defined in the bar on the left side of the figure) in which white corresponds to the earliest activation portion and purple to the latest. Between both colours a rainbow gradient shows the activation sequence. The counterclockwise circular peritricuspid movement is easily observable, although its responsibility in the arrhythmia circuit has to be verified by stimulation techniques.

Electroanatomic mapping is often a valuable tool for identifying the optimal area for ablation in many cases (Fig. 8). Both conventional fluoroscopic and electroanatomic mapping approaches have demonstrated a high acute success rate and a low recurrence rate. However, electroanatomic mapping offers the advantage of reducing fluoroscopy use and radiation exposure when compared to conventional fluoroscopy CTI ablation [108].

Di Cori *et al*. [109] in a comparative analysis between an electro-anatomical navigation system versus a conventional fluoroscopic approach in supraventricular arrhythmias found that the use of an electro-anatomical navigation system was associated with a significant reduction in total fluoroscopy time (5.5 vs 13 min) and operator radiation dose (0.8 vs 3 mSV). Of particular interest, the most remarkable absolute dose reduction was observed in cases of atrial flutter (1.3 vs 11 mSV, 88% relative dose reduction). Furthermore, atrial flutter and atrioventricular nodal reentrant tachycardia (AVNRT) were significant predictors of zero X-ray exposure at multivariate analysis with an odds ratio of 5 in the case of typical atrial flutter [105]. These findings highlight the potential benefits of employing an electro-anatomical navigation system, particularly in AVNRT and AFL cases and support its use as the choice option.

As we described before, in the study of atypical flutter cases, the use of navigation systems has demonstrated its great importance for the anatomical reconstruction of the right atrium and its anatomical barriers. These systems allow for the creation of voltage maps, enabling the localization and detection of low voltage areas and fragmented or delayed potentials, which can facilitate the identification of the macro-reentry substrate [110].

However, it is essential to emphasize that electro-anatomical mapping must always be completed with entrainment techniques, which are the most accurate evidence of the location of the reentrant circuit.

### 8.2 Anatomical Basis of CTI Ablation

**Fig. 9.**
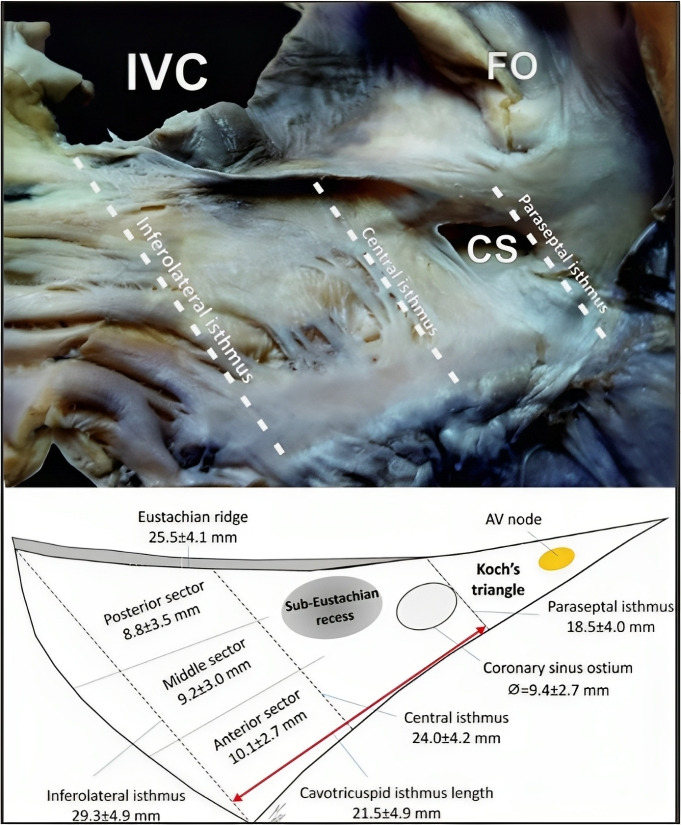
**CTI area and its proposed divisions**. Cadaveric heart image and schematic representation of the CTI and other anatomic landmarks in the right atrium. CTI, cavotricuspid isthmus; AV, atrioventricular; CS, coronary sinus; FO, fossa ovalis; IVC, inferior vena cava. Taken from: Klimek-Piotrowska W, Hołda MK, Koziej M, Hołda J, Piątek K, Tyrak K, *et al*. Clinical Anatomy of the Cavotricuspid Isthmus and Terminal Crest. PLoS ONE. 2016; 11(9): e0163383 [112].

Based on the analysis of cadaveric hearts, Cabrera *et al*. [111] proposed a three-level division of the CTI: paraseptal, inferior, and inferolateral (Fig. 9) [112]. The paraseptal isthmus, situated at the base of Koch’s triangle, also called the septal isthmus, is the shortest of the three segments but has the thickest tissue and is closest to the atrioventricular node. Conversely, the inferior isthmus, also referred to as the “central isthmus” due to its location between the other two, is the thinnest region between the inferior vena cava orifice and the tricuspid valve annulus, and therefore, it is considered the optimal ablation target.

The CTI can be divided in two different zones. An anterior zone that is typically smooth and part of the right atrium vestibule and a posterior zone that is mostly made up of fibrous and fatty tissue as it connects with the Eustachian valve [113].

Some studies with computerized tomography (CT) scan have shown that the CTI tends to change its size during the cardiac cycle, with the longest length being observed during midventricular systole. Additionally, the CTI tends to deepen during atrial contraction [114].

**Fig. 10.**
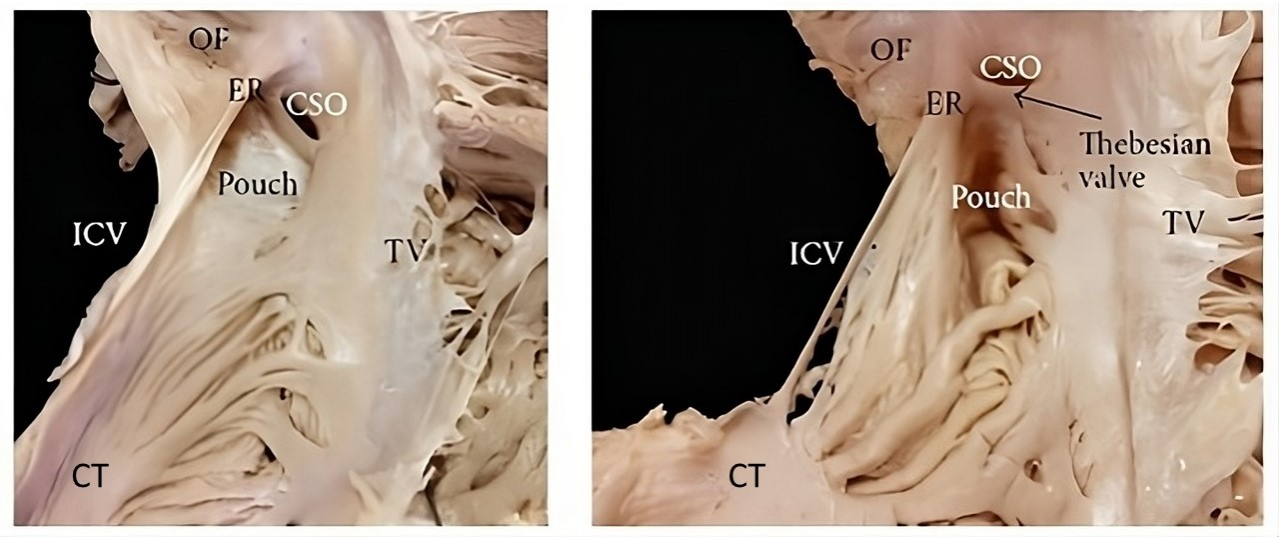
**CTI Variability**. Heart specimens that exhibit variations in the shape and structure of certain anatomical features, such as the Thebesian valve, the sub-Eustachian pouch, and the Eustachian ridge. CSO, coronary sinus orifice; ER, Eustachian ridge; ICV, inferior vena cava; OF, oval fossa; TV, tricuspid valve; CTI, cavotricuspid isthmus; CT, crista terminalis. Taken from: Sánchez-Quintana D, Doblado-Calatrava M, Cabrera JA, Macías Y, Saremi F. Anatomical Basis for the Cardiac Interventional Electrophysiologist. Biomed Res Int. 2015; 2015: 547364 [113].

In around 20% of patients, there is a sub-Eustachian (sub-Thebesian) sinus, which is a pouch-like recess located in the inferior isthmus (Fig. 10) [113].

**Fig. 11.**
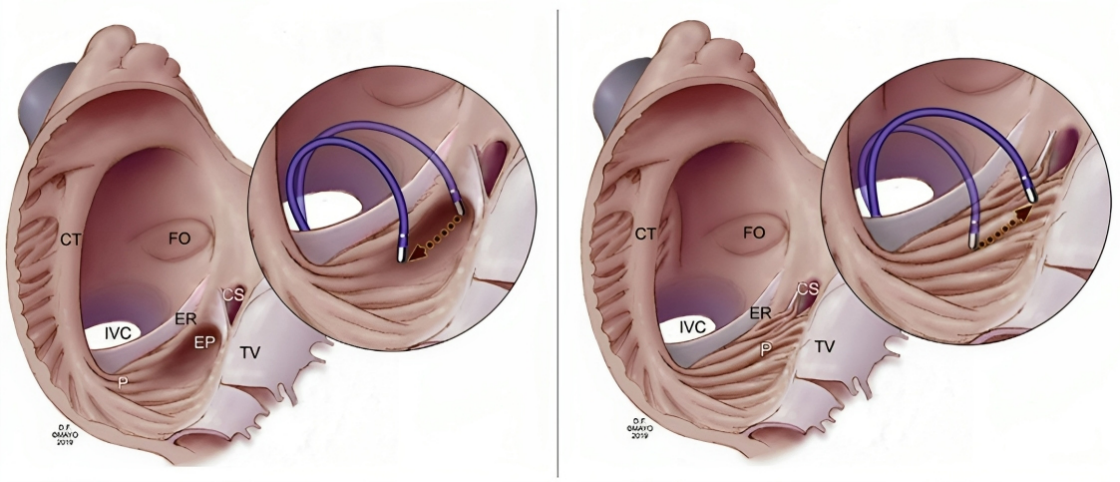
**Suggested approach for positioning the ablation catheter in patients with anatomical variants**. (Left) Lateral movement in patients with a prominent sub-Eustachian pouch. (Right) Medial movement in patients with prominent pectinate muscles. CS, coronary sinus; CT, crista terminalis; EP, eustachian pouch; ER, eustachian ridge; FO, foramen ovale; IVC, inferior vena cava; P, pectinate muscles; TV, tricuspid valve. Taken from: Christopoulos G, Siontis KC, Kucuk U, Asirvatham SJ. Cavotricuspid isthmus ablation for atrial flutter: Anatomic challenges and troubleshooting. Heart Rhythm Case Rep. 2020; 6: 115–120 [115].

This anatomical variation can lead to challenges in creating a complete line of block in this region during ablation procedures [115] For these cases, some authors recommend positioning the ablation catheter more laterally to allow better tissue contact avoiding rapid temperature and impedance rises (Fig. 11, Ref. [115]).

In patients with complex cavotricuspid anatomy, intracardiac echocardiography (ICE) can be a valuable tool and is particularly beneficial when coupled with electrophysiology procedures [116]. In a comparative study [116] evaluating atrial flutter ablation techniques, the utilization of ICE demonstrated clear advantages over the standard CTI ablation approach, with a significant reduction in both fluoroscopy time and radiation when ICE was employed. Moreover, in anotther study [117], the use of ICE during CTI ablation was associated with lower procedure time, lower X-ray exposure and lower RF energy application time.

### 8.3 CTI Ablation Procedure

CTI ablation is usually associated with moderate pain sensation, which can be even severe in some patients. Anaesthesia during catheter ablation intends to reduce patient discomfort and pain. Several approaches are being used in electrophysiology catheter laboratories, ranging from general anaesthesia to conscious sedation. Some inhaled agents such as halothane have been linked to the development of atrial tachycardias and should be avoided in this context [118]. However other commonly used drugs such as sevoflurane for general anaesthesia or intravenous agents such as propofol, midazolam or fentanyl [118, 119] have been shown to be safe without a relevant impact on tachycardia inducibility and should be the first line agents for ICT ablation.

In most cases, right femoral vein access is preferred for the ablation of the CTI. The central portion of the CTI is the narrowest and thinnest, making it the ideal target for ablation. Open irrigated tip ablation catheters are by far the most widely used, followed by solid 8-mm tip ones [4]. Open irrigated tip ablation catheters, which reduce the overheating of the tissue–electrode interface and achieve larger lesions, have demonstrated a significant reduction in both total procedure time and fluoroscopic time compared to conventional catheters. However, cautious consideration is advised for certain patients, as open irrigated catheters have been associated with potential complications such as fluid overload, symptomatic pulmonary edema, or pleural effusion, particularly in individuals with an enlarged left atrium [120]. In comparison to closed-loop irrigated RF ablation catheters, open irrigation systems have shown superior interface cooling, leading to a reduced incidence of thrombus formation and steam pops [121].

In cases where it is difficult to achieve an ICT bidirectional conduction block due to inadequate catheter contact, especially when ablation is done during arrhythmia, the use of long sheaths allows for improved stability of the catheter. Two types of sheaths are available, steerable and non-steerable. Both have the ability to enhance control over catheter manipulation, potentially offering a broader range of catheter orientations and enhanced stability. This, in turn, could lead to better tissue contact during ablation procedures resulting in more effective and precise lesion formation. The greatest reduction of procedure time and recurrent atrial arrythmias during follow-up have been reported with steerable sheaths [122, 123].

During the procedure, temperature control is maintained with a maximum limit of 45 °C and a maximum power of 50 W. The use of contact force-sensing catheters is increasing in CTI ablation [4]; however, although they seem to improve the acute efficiency of CTI ablation with lower RF times and a comparable safety as compared with conventional method, the acute success rate and long-term outcome are not significantly modified [124].

In some cases, CTI ablation can be performed using cryoablation. However, it has been shown to have a higher rate of conduction recovery through the CTI when compared to RF ablation [125].

#### 8.3.1 Ablation Techniques

The objective of isthmus-dependent atrial flutter ablation is to achieve CTI bidirectional electrical block. To accomplish this goal, various strategies are currently employed [126, 127, 128, 129, 130, 131, 132, 133, 134, 135, 136, 137, 138, 139] (Table 1, Ref. [127, 128, 129, 130, 131, 133, 134, 136, 137, 140, 141]):

**Table 1.** **CTI radiofrequency ablation techniques**.

Authors	Type of ablation	Effectiveness	Duration of fluoroscopy	Ablation time	RF aplications	Procedure time	Recurrence rate
Chen SA *et al*. 1996 [127]	Electrophysiologically-guided vs anatomical approach	93.3% vs 96.6%	42 ± 13 vs 22 ± 8 minutes	——	3 ± 1 vs 4 ± 1	181 ± 29 vs 104 ± I7 minutes	11% vs 10%
n = 60							
Shah DC *et al*. 1997 [128]	Electrophysiologically-guided approach in recurrent flutter	——–	19 ±13 minutes	12 ± 10 seconds	2 ± 2	56 ± 30 minutes	0%
n = 21							
Hall B *et al*. 2004 [129]	Anatomical vs voltage-guided approach	100% vs 100%	21 ± 10 vs 13 ± 11 minutes	5 ± 2 vs 8 ± 4 minutes	———	55 ± 33 vs 70 ± 28 minutes	19%
n = 32							
Posan E *et al*. 2007 [130]	Voltage-guided approach	———–	12.76 ± 6.57 minutes	83.8 ± 25.3 seconds	———–	68.6 ± 10.4 minutes	0%
n = 60							
Bauernfeind T *et al*. 2007 [131]	Anatomical vs voltage-guided approach	100% vs 100%	22.6 ± 10.6 vs 12.1 ± 3.8 minutes	1370 ± 1120 vs 375 ± 180 seconds	27.1 ± 21.5 vs 5.9 ± 2.4	107 ± 40 vs 68 ± 19 minutes	10% vs 10%
n = 20							
Gula LJ *et al*. 2009 [133]	Anatomical vs voltage-guided approach	100% vs 100%	23.3 ± 13.2 vs 25.0 ± 13.8 minutes	11.2 ± 7.5 vs 5.9 ± 3.3 minutes	14.2 ± 9.7 vs 7.9 ± 4.8	91.7 ± 53.0 vs 86.0 ± 50.4 minutes	3% vs 6%
n = 69							
Sato H *et al*. 2010 [134]	Anatomical vs voltage-guided approach	100% vs 100%	50.4 ± 28.3 vs 42.3 ± 21.3 minutes	1392 ± 960 vs 638 ± 342 seconds	31.7 ± 23.6 vs 13.0 ± 7.0	———–	———
n = 60							
Cheng T *et al*. 2013 [136]	Anatomical vs voltage-guided approach	100% vs 100%	18.6 ± 9.4 vs 14.8 ± 6.0 minutes	841 ± 594 vs 350 ± 319 seconds	14.0 ± 9.9 vs 5.8 ± 5.3	152 ± 58 vs 111 ± 36 minutes	5% vs 2.5%
n = 80							
Bailin SJ *et al*. 2012 [137]	Anatomical vs voltage-guided approach	100% vs 100%	28.2 ± 13 vs 27.1 ± 9.9 minutes	1194 ± 517.8 vs 451.1 ± 202.6 seconds	28.6 ± 12 vs 14.2 ± 9.8	127 ± 53 vs 119 ± 63 minutes	9 vs 0%
n = 46							
Vallès *et al*. 2023 [140]	Wave speed guided (omnipolar mapping)	92%	0 minute	349 ± 149 seconds	13	83 ± 19 minutes	3.8%
n = 26							
Maruyama M *et al*. 2006 [141]	Anatomical approach vs CTI mapping-guided ablation	100% vs 100%	28.3 ± 20.9 vs 20.8 ± 9.9 minutes	16.3 ± 11.9 vs 8.9 ± 4.4 minutes	13.8 ± 8.9 vs 7.7 ± 3.9	69.1 ± 51.0 vs 67.7 ± 30.0 minutes	5% vs 5%
n = 40							

RF, radiofrequency; CTI, cavotricuspid isthmus.

**Fig. 12.**
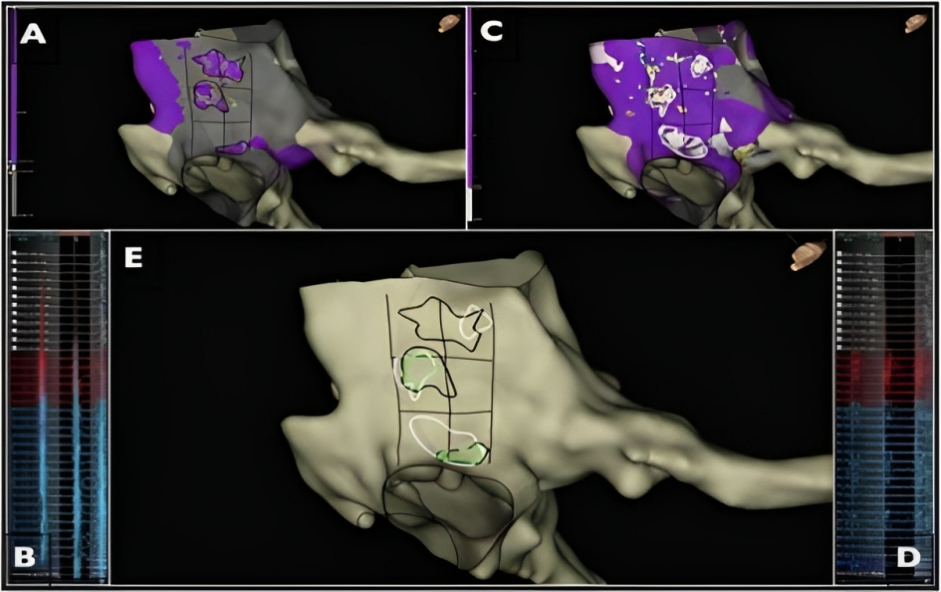
**Radiofrequency applications directed by wavefront speed and maximum voltage criteria (omnipolar mapping)**. Images from Vallés *et al*. [140] obtained by using the EnSite X mapping system and omnipolar technique showing the CTI in LAO caudal view. (A) Representation of high voltage areas, encircled in black. (B) Large electrograms in those high voltage areas. (C) Areas of slow wave speed, encircled in white. (D) Displays the fractionated EGMs in those low wave speed areas. (E) All the previous areas are displayed, highlighting confluent sites encircled in green. LAO, left anterior oblique; CTI, cavotricuspid isthmus.

(a) Creating an anatomical line of consecutive lesions in the CTI from the tricuspid valve to the inferior vena cava [126, 127] this being the most widespread technique [4]. RF ablation can be completed with point-to-point applications, keeping the distal end of the catheter stable for 45 to 60 seconds or slowly moving the catheter tip from the tricuspid valve to the inferior vena cava while delivering RF. The arrival at the entrance to the inferior vena cava is marked by a jump in the catheter, loss of the electrogram in the ablation catheter registry, and chest pain in the patient.

(b) Blocking the CTI with *electrophysiological criteria-directed point applications* based on entrainment mapping techniques [126, 127]. In this strategy, RF energy is applied to a targeted site characterized by concealed entrainment with a short stimulus-P wave interval (<40 ms) and a postpacing interval equal to the atrial flutter cycle length [126]. Alternatively, in patients with flutter recurrence after a previous ablation procedure [127], ablation is performed targeting specific areas displaying narrow or fractionated electrograms. These sites are interposed between adjacent areas with double potentials, which are considered to represent gaps.

(c) Point applications directed by maximum voltage criteria [129, 130, 131, 132, 133, 134, 135, 136, 137, 138]. This approach is based on the observation that conduction across the CTI occurs preferentially over discrete separate bundles of myocardial tissue. Accordingly, a voltage-guided ablation strategy targeting only these bundles with large amplitude atrial electrograms offers potential advantages, including interruption of conduction across the isthmus and reduction in the number of required RF applications and overall procedure time.

(d) Point applications directed by wavefront speed and maximum voltage criteria (omnipolar mapping). Omnipolar mapping [139] is a new technology available in EnSite X™ EP System (Abbott, Abbott Park, IL, USA) with the Advisor™ HD Grid (HDG) mapping catheter (Abbott), in which signals are calculated from cliques, composed of 3 unipoles and 2 orthogonal bipoles. Omnipolar electrograms provide a local signal, like a bipolar electrogram (EGM), with instantaneous information on the direction and speed of the wavefront, like a unipolar EGM, and they are displayed as beat-by-beat activation vectors. Maximum voltage can also be determined because it is truly independent of catheter–wavefront orientation. Recently, Vallès *et al*. [140] have shown that slow CTI conduction pathways can be identified by omnipolar vectors, representing unique conduction corridors in the CTI. These areas are surrounded by large muscle fibers, which are often detected on omnipolar mapping as high voltage areas (Fig. 12, Ref. [140]). Ablation in areas of slow conduction would achieve CTI bidirectional block in more than 92% of patients, suggesting they should be targeted preferentially. Possible advantages of this approach could include less procedure and fluoroscopy time, and a reduction in RF burden.

Electro-anatomical navigation systems offer substantial support in all three strategies, but their benefits are especially pronounced in the last three. By allowing for the precise identification and marking of critical structures, these systems enable efficient navigation during the procedure and significantly reduce radiation exposure, with the potential to perform the procedure entirely free of fluoroscopy [142].

#### 8.3.2 Acute Success Criteria for Atrial Flutter Ablation

When performing CTI ablation in atrial flutter, the reversion to sinus rhythm during RF application does not guarantee the success of the procedure. It is important to continue the application until the ablation line is completed and reaches the inferior vena cava.

After finishing the CTI ablation, bidirectional conduction block should be confirmed by pacing on both sides of the ablation line. Various criteria based on analysis of the atrial activation sequence on the both sides of the ablation line, characteristics of local electrograms recorded in that area, consideration of the temporal relationship between the P wave in lead V1 and the second component of the local atrial electrogram along the ablation line [7], the paced PR interval [8], and conduction times between electrograms recorded during atrial pacing on the ablation line have been proposed for this purpose [143, 144, 145, 146].

Double potentials separated by an isoelectric line of ≥30 ms should be recorded at the ablation line during low lateral right atrium or coronary sinus ostial pacing. Gaps in this line (i.e., sites of persistent conduction) are usually localized by fractionated potentials centered on or occupying the isoelectric interval of adjacent double potentials [146].

**Fig. 13.**
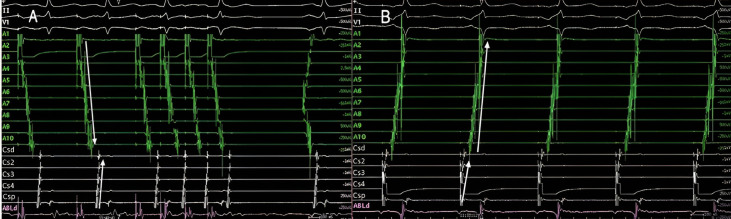
**CTI block and non-inducibility evaluation**. (A) Pacing from the low anterolateral right atrium (dipole A1) demonstrating ICT clockwise block. Activation of the RA is performed along the tricuspid annulus (A1-A10) until it reaches proximal coronary sinus (CSp). The administration of 3 extrastimuli does not induce any tachycardia. (B) Pacing from the coronary sinus os reaches the inferior septum (A10) and the activation wavefront is propagated toward A10-A1, demonstrating CTI counterclockwise block. CTI, cavotricuspid isthmus; RA, right atrium.

To confirm counterclockwise CTI block, pacing is performed from one of the dipoles of the duodecapolar catheter located on the lateral part of the CTI or directly with the ablation catheter (Fig. 13A). Conversely, to confirm clockwise CTI block, pacing is performed from the ostium of the coronary sinus (Fig. 13B). It is important to use relatively slow pacing frequencies to avoid rate-dependent CTI block [147].

Another interesting technique used to confirm bidirectional block is differential pacing using the distal and proximal bipoles of a quadripolar catheter placed close to the ablation line [148].

Local fractionated potentials are commonly observed in the right atrium and often indicate the presence of conducting gaps. However, in some cases, these potentials may also signify bystander zones of slow conduction that occur in complete isthmus block. During unidirectional activation of the isthmus, the electrograms recorded along the ablation line provide valuable insights into the activation pattern in its proximity. Specifically, the initial component of the electrogram reflects activation at the ipsilateral border of the ablation line, while the terminal component represents activation at the contralateral border. Differential atrial pacing is employed to assess whether components of the local potentials recorded from the ablation line are produced by a penetrating wavefront of persisting isthmus conduction or by wavefronts colliding on either side of a complete line of block [148]. In this regard, changing the pacing site further away from the ablation line can be useful in predicting the presence of conduction gaps. When a wavefront passes anterogradely through a gap in the ablation line, both components of the electrogram for double potentials, or all components for triple potentials, are delayed similarly. However, if there is a complete isthmus block, the wavefront bypasses the ablation line, resulting in an increase in the timing of the initial electrogram component, and either no change or a decrease in the timing of the terminal component.

The differential pacing response for complete isthmus block showed a sensitivity of 100%, a specificity of 75%, a negative predictive value of 94%, and a positive predictive value of 100% [148].

**Fig. 14.**
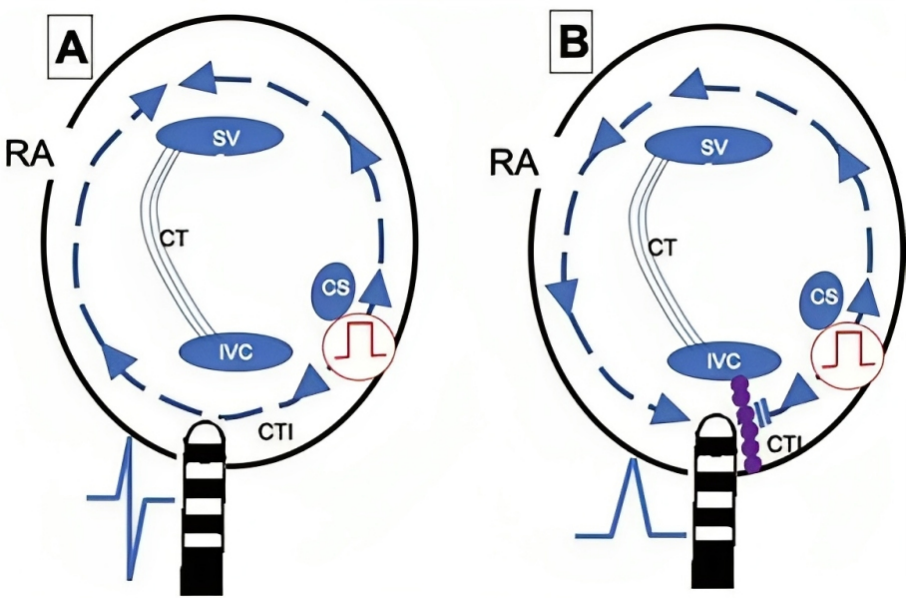
**Usefulness of unipolar electrograms to detect CTI block**. (A) Unipolar electrogram recorded at low anterolateral right atrium while pacing from CSos with R/S morphology suggesting permeable CTI. (B) Unipolar electrogram recorded after CTI ablation, changing to R morphology suggesting termination of wavefront against a line of block. Purple dots represent radiofrequency applications. CSos, coronary sinus ostium; CT, crista terminalis; IVC, inferior vena cava; RA, right atrium; SV, superior vena cava; CTI, cavotricuspid isthmus; CS, coronary sinus. Courtesy of Dr. J. Villacastin [149].

Villacastín *et al*. [149] proposed a simple approach to confirm bidirectional CTI block post ablation, by analyzing changes in unipolar electrograms of the right atrium. The technique involves obtaining unipolar electrograms before and after CTI ablation at the low anterolateral right atrium during coronary sinus pacing. In patients with clockwise and counterclockwise CTI block, changes in the morphology of the unipolar electrogram from RS, rs, or QS to R or Rs were observed. An unchanged unipolar electrogram after ablation was obtained in a patient in whom CTI block was not achieved. The unipolar electrogram was able to correctly predict 100% of cases with clockwise CTI block and 89% of cases with counterclockwise CTI block (Fig. 14, Ref. [149]).

Finally, there are 2 potential pitfalls that are always worth considering when evaluating CTI after applications. Firstly, the evaluation of isthmus block response to pacing is dependent on several fundamental characteristics, including the anisotropic conduction properties of the crista terminalis, functional block, impulse conduction velocity, and direction of propagation [150]. Consequently, there are two important phenomena that need to be considered when verifying CTI block during coronary sinus (CS) pacing. The first phenomenon involves pseudoconduction across the CTI due to the absence of a functional conduction block along the crista terminalis. In this case, conduction across the crista terminalis would give the appearance of clockwise conduction over the CTI. Failing to recognize pseudoconduction across the CTI may lead to unnecessary additional ablation lesions [18].

Secondly, pseudoblock is another crucial aspect to take into account. This term refers to the potential activation of the right atrium across Bachmann’s bundle when pacing in the CS, particularly in cases where the conduction properties of the CS to the right atrium are compromised [150]. This could result in a right atrial activation pattern consistent with a false unidirectional pseudoclockwise block in the CTI.

After ablation, programmed atrial pacing administrating up to 3 extra stimuli are recommended, even during isoproterenol perfusion [151]. In some cases, adenosine [152] can be used to assess bidirectional CTI block. These pharmacological agents can help identify areas of residual conduction and ensure the success of the procedure.

Transient CTI block can occur, so a waiting period of 20–30 minutes is necessary to confirm the success of the procedure. In up to 15% of cases, conduction through the CTI can be re-established during long-term follow-up, even in the absence of flutter recurrence [125].

### 8.4 Ablation of Atypical Flutters

In general, atypical flutter refers to macroreentrant atrial tachycardias in which the wavefront does not propagate around the tricuspid annulus and the CTI is not part of the reentrant circuit [153, 154]. However, it is important to keep in mind that, even in complex situations such as corrected Ebstein’s anomaly or tricuspid prosthesis or rings, the presence of CTI-dependent flutter with atypical morphology is not rare [28, 155]. Structural abnormalities such as atriotomy scars, patch closure of atrial septal defects, repaired congenital heart disease, or suture lines after cardiac surgery can act as barriers to the conduction of electrical impulses and promote the development of a reentrant circuit [154, 155]. Ablation of atypical flutters offers worse long-term results compared to typical flutters, with success rates ranging from 70% to 80% and a higher likelihood of recurrence during long-term follow-up [156]. As mentioned previously, ablation strategies in these patients focus on defining the reentry circuit and subsequently creating an ablation line in an area of the circuit between anatomical barriers or scars or identifying slow conduction isthmuses located between areas of inexcitable tissue, where low-amplitude potentials with double or fractionated electrograms are characteristically observed, and which are susceptible to focal ablation [101]. In the case of a CTI dependent flutter, in patients with corrected Ebstein’s anomaly, a tricuspid annuloplasty ring or a prosthetic tricuspid valve, CTI ablation may require RF applications from the ventricular side of the valve to target atrial tissue rendered inaccessible as a result of tricuspid valve surgery [155].

### 8.5 Complications

Complications related to the ablation procedure are infrequent and typically limited to vascular access. Serious and potentially life-threatening complications have been described in 0.5–0.7% of cases [157, 158]. Some of the described serious complications include atrioventricular block, stroke, acute myocardial infarction resulting from injury to the right coronary artery, or cardiac perforation. In cases where left atrial flutter ablation is performed, the risk of complications such as arterial embolism, aortic injury, mitral valve injury, or atrioesophageal fistula should also be considered [157, 158, 159].

A rare, but relevant, complication with open-irrigated catheters during RF ablation is the potential discrepancy between electrode temperature and tissue temperature, where tissue temperatures may surpass catheter tip temperatures significantly. Steam pops refer to the audible sound produced by intramyocardial explosion when tissue temperature reaches 100 °C. This phenomenon results in the formation of gas within the myocardium, which cannot easily diffuse away from the heated zone, leading to an increase in local pressure. While steam pops are relatively uncommon, occurring in only 0.1% to 1.5% of cases, they pose a potentially severe complication of the procedure and have been associated with embolic stroke, cardiac perforation, and ventricular septal defect [160]. Clinical strategies to reduce the risk for steam pops would include immediate reduction of power if a large impedance decrease is detected, power titration to 50 W (compared with fixed maximum-power delivery) and careful attention to lesion formation using ICE [160].

In the latest ablation registry of Spain, there were 22 reported complications out of 3766 procedures (0.6%), with the majority of cases related to vascular access. Additionally, there were 2 cases of pericardial effusion and 1 case of myocardial infarction [4].

### 8.6 CTI Ablation Results

The rate of atrial flutter recurrence after a successful procedure is typically ≤10% [125]. However, this rate may be increased (18%) in patients with a history of surgical correction for acquired heart disease (coronary and valvular heart disease) and congenital heart disease (such as ostium secundum atrial septal defect and complex cases). Additionally, female gender and severe left ventricular dysfunction [28, 161] have been associated with higher recurrence rates. However, in those patients who experience recurrence, a second cavotricuspid isthmus ablation is usually effective in achieving long-term success.

A meta-analysis of 48 studies conducted between 1996 and 2015 evaluated the results of RF ablation in patients with atrial flutter [162]. The analysis revealed that de novo atrial fibrillation occurred in 23% of patients during a 2.5-year follow-up period. Patients with a history of paroxysmal atrial fibrillation had a higher recurrence rate, up to 52%.

The appearance of atrial fibrillation after atrial flutter ablation seems to be influenced by the previous presence of this arrhythmia. In a study that included 100 patients, 29 of whom had previously experienced an episode of atrial fibrillation, 36.4% experienced an episode of atrial fibrillation during a 15-month follow-up during a 15-month follow-up [163]. However, in a similar study of patients without a history of atrial fibrillation, this arrhythmia was only recorded in 12.9% of individuals during a 19-month follow-up [164]. Most patients who develop atrial fibrillation after ICT ablation do so within the first 6 months.

In patients with coexisting atrial flutter and atrial fibrillation, some working groups have proposed performing a combined ablation of the CTI and pulmonary veins [165, 166]. To determine the optimal approach for patients with both arrhythmias, the APROVAL study [167], a randomized, single-blind clinical trial including 360 patients, was conducted. Patients were randomized into two groups: those who underwent pulmonary vein ablation (with or without additional CTI ablation) and those who underwent only CTI ablation. After a 22-month follow-up, the first group had a significantly higher rate of absence of arrhythmias without antiarrhythmic treatment (64% vs 19%). Interestingly, this finding was consistent in both subgroups, those who received atrial fibrillation ablation with or without additional CTI. These results are consistent with other smaller studies, which suggest that pulmonary vein potentials may act as triggers for both atrial fibrillation and flutter episodes [166].

To date, there are no proven guidelines to define which patients with CTI ablation and without a previous history of AF require a more extensive approach that includes pulmonary vein isolation. Studies in which patients have been empirically randomized to CTI ablation with or without pulmonary vein isolation show a better prognosis in those in whom both substrates have been targeted [168].

Several factors have been identified as predictors of the development of new atrial fibrillation in patients with a prior CTI ablation. These include mitral regurgitation [169], inducibility of sustained atrial fibrillation [170], and left atrial diameter [171]. For patients with a history of atrial fibrillation, body mass index [171] and left ventricular ejection fraction [169] have also been identified as predictors of the development of atrial fibrillation. The inducibility of AF by stimulation during the isthmus ablation has shown a strong predictive value of AF risk after CTI ablation, with an odds ratio in the prospective studies of 5.52 [172], although with the disadvantage of not having that information available prior to the procedure. There are no randomized trials that address whether this parameter or any other derived from other techniques (e.g., imaging) can reliably stratify patients who require a more aggressive procedure or closer follow-up.

In the specific case of patients with atrial fibrillation developing typical atrial flutter while they are under treatment with class IC antiarrhythmic drugs, IC class flutter, multiple small studies have suggested a hybrid therapy for maintaining sinus rhythm in these patients, consisting of CTI ablation and continued treatment with antiarrhythmic drugs. This approach has demonstrated considerable effectiveness in maintaining sinus rhythm, with some studies reporting long-term arrhythmia-free survival rates of 80–90% [173]. Therefore, an attempt of isolated ICT ablation as a first choice would be admissible, especially in patients in whom flecainide and anticoagulant treatment can be maintained (see anticoagulation section).

## 9. Conclusions 

The pathophysiological mechanisms that initiate atrial flutter remain unclear. Although current research has primarily focused on developing effective ablation techniques, it is essential to continue exploring the electrophysiological, ultrastructural, and pharmacological pathways underlying arrhythmia development to devise targeted preventive strategies.

From the initial X-ray guided linear CTI ablation, supported by the electro-anatomical navigation systems, several alternatives have arisen in the last decade, such as electrophysiological criteria-directed point applications based on entrainment mapping, applications directed by maximum voltage criteria or by wavefront speed and maximum voltage criteria (omnipolar mapping). Rigorous verification of persistent CTI bidirectional blockade based on various stimulation techniques is mandatory.

Finally, a remaining major challenge is identifying patients who are at risk of developing post-ablation atrial fibrillation. Discriminating between individuals who will experience a complete arrhythmia cure and those who will develop AF after flutter ablation is essential given the important prognostic and therapeutic implications.
